# Transport Stress Induces Skin Innate Immunity Response in Hybrid Yellow Catfish (*Tachysurus fulvidraco*♀ × *P. vachellii*♂) Through TLR/NLR Signaling Pathways and Regulation of Mucus Secretion

**DOI:** 10.3389/fimmu.2021.740359

**Published:** 2021-10-12

**Authors:** Tao Zheng, Zhuo Song, Jun Qiang, Yifan Tao, Haojun Zhu, Junlei Ma, Pao Xu

**Affiliations:** ^1^ Wuxi Fisheries College, Nanjing Agricultural University, Wuxi, China; ^2^ Key Laboratory of Freshwater Fisheries and Germplasm Resources Utilization, Ministry of Agriculture and Rural Affairs, Freshwater Fisheries Research Center, Chinese Academy of Fishery Sciences, Wuxi, China

**Keywords:** transport stress, hybrid yellow catfish, immune, toll-like receptors, NOD-like receptors

## Abstract

The transport of live fish is a necessary step for commercial production. The skin of teleost fish is the first non-specific immune barrier against exogenous stimuli, and it plays an important protective role under transport stress. Thus, the aim of this study was to explore the skin responses to transport stress in hybrid yellow catfish (*Tachysurus fulvidraco*♀ × *Pseudobagrus vachellii*♂) through transcriptome and biochemical analyses. Water samples were collected during a simulated transport treatment. Biochemical indexes and/or gene expression in blood, skin, and mucus in fish in control groups and transport-stress groups (0 h, 2 h, 4 h, 8 h, 16 h) were assayed. The levels of total ammonia–nitrogen and nitrite–nitrogen in the water increased with increasing transport time. Comparison of skin transcriptomes between the control group and the group subjected to 16 h of transport revealed 1547 differentially expressed genes (868 up-regulated and 679 down-regulated). The results of the transcriptome analysis were validated by analyses of the expression levels of selected genes by qRT-PCR. The results indicated that the toll-like receptors and nod-like receptors signaling pathways mediate the skin’s immune response to transport stress: *tlr9*, *mfn2*, and *ikbke* were significantly up-regulated and *nfkbia* and *map3k7cl* were significantly down-regulated under transport stress. With increasing transport time, lysozyme activity and the immunoglobulin M content in skin mucus first increased and then decreased. The number of mucous cells peaked at 8 h of transport stress, and then decreased. The mucus cells changed from types II and IV to types I, II, III, and IV. The amounts of red and white blood cells and the levels of hemoglobin and hematocrit first increased and then decreased during 16 h of transport stress. Together, the results showed that the skin responds to transport stress by activating the immune signaling pathway and regulating mucus secretion. These findings have important biological significance for selecting strains that tolerate transport, as well as economic significance for optimizing the transport conditions for scaleless fish.

## 1 Introduction

The transportation of live fish is an essential step to meet the demand for fish in different regions. The extent of harm caused by transport depends on the duration and severity of the stress, as well as the health status of the fish (1). During transportation, fish are in an enclosed environment, and their metabolic wastes affect water quality parameters such as the pH and the concentrations of ammonia, nitrite, and carbon dioxide (2–5). The accumulation of total ammonia–nitrogen and nitrite–nitrogen in water can lead to transportation stress (6). With prolonged transit time, transport stress can cause physiological dysfunction in fish, and consequently diminish their resistance to pathogens, even resulting in death (7–10). Studies have shown that transport stress affects the innate immunity of fish (9).

For teleost fish, the skin is a key component of innate immunity. It ensures that they can survive and adapt to the environment (11, 12). Skin with its outermost mucus separates the individual from environment and is the crucial interface for communication and contact with external factors (13). The skin and its mucus often be used to study the immune response of fish under stress in aquaculture. In sea bream (*Sparus aurata* L.), chronic crowding stress was found to reduce the barrier function of damaged skin and suppress the local immune response to scale removal (14). In *Epinephelus coioides*, the skin showed a strong immune response to the invasion of *Cryptocaryon irritans*, and genes encoding Toll-like receptors (*tlr1*, *tlr*2, *tlr*5 and *tlr5s*) were found to be significantly up-regulated (15). As a skin appendage, mucus is produced by dynamic secretory cells in the epidermis. The change of its composition and rheological properties plays an important role in regulating mucosal immunity in fish (16). The different proteases found in fish surface mucus are involved in innate immunity (17). Among these proteases, immunoglobulin M (IgM) in skin mucus is the first responder to foreign invaders, and mucosal lysozyme (LZM) is a antimicrobial enzyme with antibacterial, antimetastatic, antiviral, and anti-inflammatory properties (18). Mucosal cells and the composition of mucus can be affected by exogenous factors (such as stress, acid, and infections) and endogenous factors (such as sex and developmental stage) (19–21). In sea bass (*Laeolabrax japonicus*), vigorous proliferation of skin mucus cells was observed under prolonged hypoxia and high nitrate levels (22). In large yellow croaker (*Larimichthys crocea*), the cortisol, malondialdehyde and IgM levels in skin mucus increased first and then decreased under high-temperature (23).

Hybrid yellow catfish (*Tachysurus fulvidraco*♀ × *Pseudobagrus vachellii*♂) shows good resistance and a fast growth rate, it has a short breeding cycle and a high meat content, and it is widely farmed (24). However, hybrid yellow catfish seem to be highly susceptible to transport stress. In recent years, high-throughput sequencing technology has been used to analyze the responses of aquatic animals to various stresses. Using this technology, researchers have discovered a series of molecular changes that occur under transport stress. For example, a total 1275 differentially expressed genes (DEGs) were identified in the liver of zebrafish (25) and 1000 DEGs were identified in the muscle of rainbow trout under transport stress (26). Those genes were found to be involved in metabolic processes, cellular homeostasis, and the immune response, indicating that these processes rapidly change under transport stress. Thus, transcriptome analysis is a useful tool to explore the responses of fish to transport stress. However, data on skin transcriptome responses of hybrid yellow catfish to transport stress are lacking. Here, we used transcriptome analyses to identify differentially expressed genes (DEGs) in yellow catfish under transport stress. Several randomly selected DEGs were verified by qRT-PCR to confirm the reliability of the RNA-Seq results. We also analyzed skin sections and mucus biochemical factors, including IgM content and LZM activity, in fish under transport stress. Our results revealed significant changes in gene expression levels in the skin and mucus attributes of yellow catfish under transport stress. These results shed light on the responses and regulatory mechanisms in skin of fish under transport stress.

## 2 Materials and Methods

### 2.1 Fish and Experimental Design

Juvenile yellow catfish with an average length (± SD) of 5.06 ± 0.82 cm and an average mass ( ± SD) of 7.06 ± 1.30 g were obtained from the Freshwater Fisheries Research Centre (Yixing, China). The fish were fed with commercial feed (crude protein 38.0%, crude fat 6%) at 2% (w/w) of their body weight twice per day for 7 days in a recirculating aquaculture system (temperature 26 ± 3°C DO > 5.5 mg/L; ammonia nitrogen and nitrite < 0.01 mg/L; pH 7.5 ± 0.3) before experiments. The experimental fish were subjected to a simulated transport treatment after starvation for 24 h. For the simulated transport treatment, 30 fish were placed in each double-layered plastic nylon bag (40 × 82 cm) with about one-third of the volume occupied by freshwater and two-thirds of the volume occupied by pure oxygen (5). The transport bags were sealed with rubber bands and placed on an analog transporter (simulated automotive shaker; Changzhou, China). The vibration frequency was set at 100 RPM (revolutions per minute) according to the method of Wu et al. (5). We established five transport groups (0 h, 2 h, 4 h, 8 h, and 16 h) and one group that was allowed to recover for 96 h after 16 h of transport stress. Each group had three replicates. Control fish of each group were obtained from the recirculating aquaculture system (temperature 26 ± 3°C DO > 5.5 mg/L; ammonia nitrogen and nitrite < 0.01 mg/L; pH 7.5 ± 0.3). To monitor recovery after 16 h of transport stress, the fish in the 96h recovery (96hr) group were kept under the same conditions as those in the control group.

### 2.2 Sample Collection

At each time point, 12 individual fish were randomly selected from each group and were anesthetized immediately with 200 mg/L MS-222 (27). Water samples were collected at the same time. Blood samples were taken from the tail vein using a 2.5-mL syringe to use for blood cell analysis. Before collecting mucus, water was gently removed from the fish. The fish were then placed in a sterile bag for 1 minute to collect the mucus (28). The skin mucus was immediately homogenized with Tris-buffered saline (TBS, pH 8.0, 50 mM Tris HCl, 150 mM NaCl) on ice and the mixture was centrifuged at 12000 g and 4°C for 15 min (29). The supernatant was collected and stored at −80°C until further analysis. Skin was collected from the left side of each fish behind the dorsal fin, and was separated into two subsamples: one subsample was used for histological analysis, and the second subsample was frozen in liquid nitrogen and then stored at −80°C until transcriptome analysis.

### 2.3 Water Quality Detection

We used a portable DO meter (YSI 556, Yellow Springs Instruments, Yellow Springs, OH, USA) to determine the DO content. According to Qiang et al. (30), we determined total ammonia–nitrogen (TAN) and nitrite–nitrogen (NO_2_-N) by spectrophotometric methods.

### 2.4 Blood Analysis

White blood cells (WBC); hemoglobin (HGB); hematocrit (HCT); and red blood cells (RBC) were quantified using a Blood cell analyzer (Mindray, bc-5300, Shenzhen, China) according to the manufacturer’s instructions. All of the reagents were obtained from Shenzhen MINDRAY BioMedical (Shenzhen, China).

### 2.5 Mucus Analysis

The activity of LZM was assessed using a commercially available kit (Jiancheng Institute of Biotechnology). The IgM in skin mucus was detected using enzyme-linked immunoassay (ELISA) (Shanghai Lengton Bioscience Co., Ltd.) as described by Guardiola et al. (29, 31).

### 2.6 Histological Analysis

The skin samples were fixed in Bouin’s solution for 24 h and then transferred into 70% ethanol solution. Samples were soaked in water for a few hours, and then gradually dehydrated for 1 h with an ethanol gradient (70% ethanol, 80% ethanol, 95% ethanol, 95% ethanol, anhydrous ethanol, anhydrous ethanol). The samples were immersed in a mixture of xylene and anhydrous ethanol (v/v 1:1) for 1 h, then immersed in xylene I and xylene II for 2 h separately to improve transparency. The samples were embedded in paraffin using a Leica EG 1150 H embedding machine. The embedded samples were sliced into 7-µm sections using a Leica RM 2255 microtome. The sections were developed using a Leica HI 1210 profiling machine, dried at room temperature, then baked at 60° for 1 h. The sections were then immersed in xylene I for 15 min and xylene II for 15 min before immersion in 100%, 95%, 90%, 80%, and 70% alcohol solution (5 min each). After washing for 3 min and dewaxing, the sections were stained with alcian blue-periodic acid-Schiff (AB-PAS). The sections were sealed with neutral gum and observed and photographed under a NIKON Eclipse Ci microscope (NIKON, Tokyo, Japan) and NIKON digital sight DS-FI2 imaging system, respectively.

### 2.7 RNA Sequencing and Quantitative Real-Time PCR Analysis

#### 2.7.1 mRNA Library Construction and Sequencing

Total RNA was extracted from the skin of fish in the control group and the 16-h transport group using Trizol reagent (Invitrogen, Carlsbad, CA, USA), according to the manufacturer’s instructions. The quantity and purity of RNA were checked using a Bioanalyzer 2100 and RNA 6000 Nano Lab Chip Kit (Agilent, Palo Alto, CA, USA) with RIN >7.0. The poly (A) mRNA was isolated from approximately 10 µg RNA with poly-T oligo-attached magnetic beads (Invitrogen). The mRNA was fragmented into small pieces using divalent cations. After purification, the cleaved RNA fragments were reverse-transcribed to build the final cDNA library according to the protocol of the mRNA Seq sample preparation kit (Illumina, San Diego, CA, USA). The average insert size was 300 bp ( ± 50 bp) for the cDNA libraries. Following the manufacturer’s recommended protocol, the transcriptomes were sequenced, generating paired-end reads of 150bp length on the IlluminaHiseq4000 platform at LC Sciences (Houston, TX, USA) using the Illumina paired-end RNA-seq approach.

#### 2.7.2 Quality Control, RNA-Seq Reads Mapping, and DEGs Analysis

Unqualified raw data were filtered using Cutadapt to obtain clean data. We aligned reads from the control group and 16-h transport group to the UCSC (http://genome.ucsc.edu/) *Homo sapiens* reference genome using the HISAT package, which initially removed a portion of the reads based on quality information accompanying each read, and then mapped the reads to the reference genome. For each sample, the mapped reads were assembled using StringTie to reconstruct a comprehensive transcriptome using perl scripts. Then, all the transcriptomes were merged to reconstruct a comprehensive transcriptome using perl scripts. After the final transcriptome was generated, StringTie and edgeR were used to estimate gene transcript levels. StringTie was used to calculate the FPKM values, which were indicative of the mRNA levels. The DEGs between control and 16-h transport stressed fish were selected based on log2 (fold change) >1 or log2 (fold change) <-1 and statistical significance of *p* < 0.05 by the R package. Functional analysis of DEGs was conducted using tools at the GO (Gene Ontology) and KEGG (Kyoto Encyclopedia of Genes and Genomes) databases. The results of GO and KEGG enrichment analysis are presented as a scatter diagram, which was constructed using GGplot2. Based on the KEGG pathways, we identified the DEGs. We focused on identifying immune-related pathways in the skin of hybrid yellow catfish under transport stress and selected key genes for qRT-PCR verification.

#### 2.7.3 Validation of DEGs in Skin by qRT-PCR

We verified the transcriptional patterns of DEGs under transport stress by qRT-PCR. Total RNA was extracted from skin using the RNAiso Plus kit (TaKaRa, Dalian, China). The quality and concentration of RNA were determined from the absorbance values at 260 nm and 280 nm (A260/A280 ratio, 1.9–2.1). Then, cDNA was synthesized by PrimeScript™ RT reagent kit with gDNA Eraser (TaKaRa). The relative transcript levels of genes associated with TLRs and NLR signaling pathways were determined by qRT-PCR using the following program: one cycle at 95°C for 30s, then 40 cycles at 95°Cfor 5 s, and 60–63°C for 1 min. The amplification mixture (20 μL) comprised 2 μL cDNA, 10 μL TB Green Premix Ex Taq II (Takara), 0.5 μL each primer, and 7 μL ddH_2_O. *β*-actin was the reference gene. The relative transcript levels were determined by the 2^−ΔΔCT^ method. The primers used are shown in **Table 1**.

**Table 1:** The specific primer sequences for qPCR in this study.

Gene	Primer sequence (5’-3’)	Efficiency%	Amplicon size	GenBank number
*tlr9*	F: GGGCAGGACACAAGGGTTAT R: CCTCCAGACAAAGCCGGAAT	99.639	171	XM_027167061.1
*mfn2*	F: CGGCATCTTTGAGCAACTCG R: TGCCACCTTGGACAGGTAAC	105.677	139	XM_027166944.1
*txnipb*	F: CTGAACTTACGACTCCCGCC R: CGGGAGCAGGAGTTCTCAAA	101.047	160	XM_027148810.1
*nfkbia*	F: CACGTTTGGTGGATCAGTGC R: TGTCCGCTGTAGTTGTGGAC	101.797	139	XM_027154956.1
*map3k7cl*	F: AACAGAAATCGCTCTGCTGGA R: TGACGAGGGTATGGTTCTCCT	97.141	137	XM_027171703.1
*ikbke*	F: GAGATCCAGGCGAACCCAAA R: AGGGAGTCCGAATGCGTTTT	104.487	105	XM_027144349.1
*β-actin*	F: GGATTCGCTGGAGATGATG R: TCGTTGTAGAAGGTGTGATG	99.268	221	XM_027148463.1 (32)

### 2.8 Statistical Analysis

Differences within the same treatment group at different times were detected by one-way analysis of variance (ANOVA). Data are presented as mean ± standard error of the mean (SEM). Differences were analyzed by Duncan’s multiple range test using SPSS 26.0 (SPSS Inc., Chicago, IL, USA). The independent-samples t test was used to detect significant differences between the control group and transport group at each time point. The level of significance was *P <*0.05.

## 3 Results

### 3.1 Water Quality Parameters During Simulated Transport

**Figure 1:**
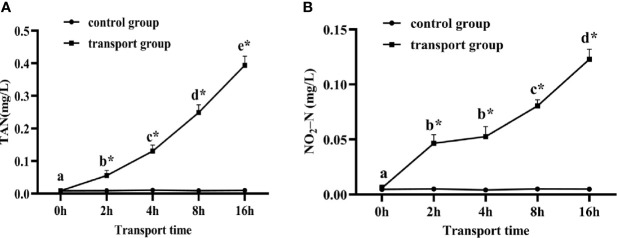
Changes in total ammonia nitrogen (TAN) **(A)** and nitrite-nitrogen (NO_2_-N) **(B)** levels in water of fish during transport. stress. Values are mean ± SD (*n*=12). Asterisk (*) indicates significant difference (*P* < 0.05) between control group and transport group. Different small letters indicate significant differences (*P* < 0.05) among sampling times in the same group.

As shown in **Figure 1**, there was no significant change in TAN and NO_2_-N in the water of the control groups. The TAN and NO_2_-N contents differed significantly between the control and the transport groups at all times except for 0 h. In the transport groups, TAN (*P*< 0.05; **Figure 1A**) levels clearly increased from 0 h onwards. The NO_2_-N (*P* < 0.05; **Figure 1B**) levels increased during transport, but were not significantly different between 2 h and 4 h. The DO levels were higher than 22 mg/L in the transport groups during the simulated transport treatment.

### 3.2 Changes in Blood Parameters in Fish During Simulated Transport

**Figure 2:**
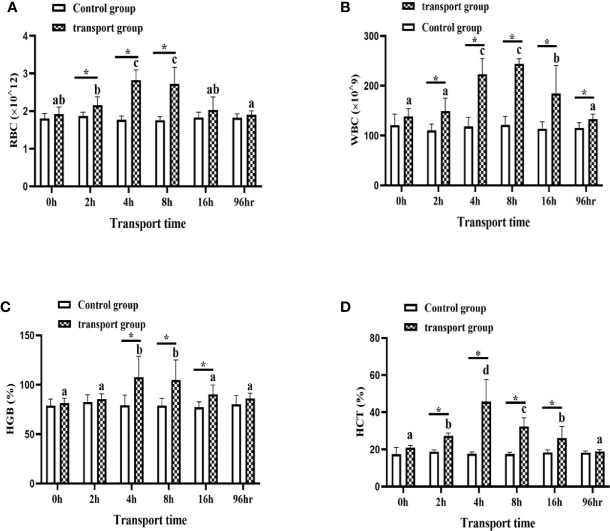
Amounts of white blood cells (WBC) **(A)** and red blood cells (RBC) **(B)** and levels of hemoglobin (HGB) **(C)** and hematocrit (HCT) **(D)** in transported and recovery groups. Values are mean ± SD (*n*=12). Asterisk (*) indicates significant difference (*P* < 0.05) between control group and transport group. Different small letters indicate significant differences (*P* < 0.05) among sampling times in the same group.

For control groups, there was no significant difference in RBC, WBC, HGB, and HCT among the sampling times. For transport groups, the RBC, WBC and HGB were significantly up-regulated at 4 h and 8 h compared with 0 h, and significantly down-regulated at 16 h compared with 4 h and 8 h but still higher than at 0 h (*P*<0.05; **Figures 2B, C**). The HCT were significantly increased at 2 h and 4 h of transport compared with 0 h, peaked at 4h and then by 8 h and 16h they decreased to a level similar to that at 2h (*P*<0.05; **Figures 2A, D**). In addition, compared with the control groups, the transportation groups showed higher RBC, WBC, HGB, and HCT levels at all sampling times except for 0 h. In the 96h recovery group, the amounts of RBC and the levels of HGB and HCT were not significantly different from those in the control group. However, the amount of WBC was higher than that in the control group, but lower than that in the 16 h transport stress group (*P*<0.05; **Figure 2B**).

### 3.3 Mucus Biochemical Analyses and Skin Histological Analyses

**Figure 3:**
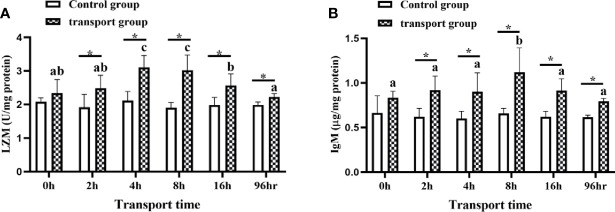
Lysozyme (LZM) activity **(A)** and immunoglobulin M (IgM) levels **(B)** in skin mucus of transported and recovery groups. Values are mean ± SD (*n* =12). Asterisk (*) indicates significant differences (*P* < 0.05) between control group and transport group. Different small letters indicate significant differences (*P* < 0.05) among time points within the same group.

The LZM activity and IgM content in mucus did not differ among time points in the control groups. However, the LZM content differed between the control groups and the transportation groups at all sampling times except for 0 h. The IgM content also differed significantly between the control groups and transportation groups at all sampling times except for 0 h. In the transportation groups, LZM activity was higher at 4 h and 8 h than at 0 h and 2 h of transport (*P*<0.05; **Figure 3A**) but significantly decreased by 16 h of transport. The IgM content showed a slight upward trend at 2 h and 4 h and was clearly higher at 8 h than at 0 h of transport, but decreased at16h (*P*<0.05; **Figure 3B**). The LZM and Igm levels in the 96h recovery group were higher than those in the control group but lower than those in the 16h transport stress group.

**Figure 4:**
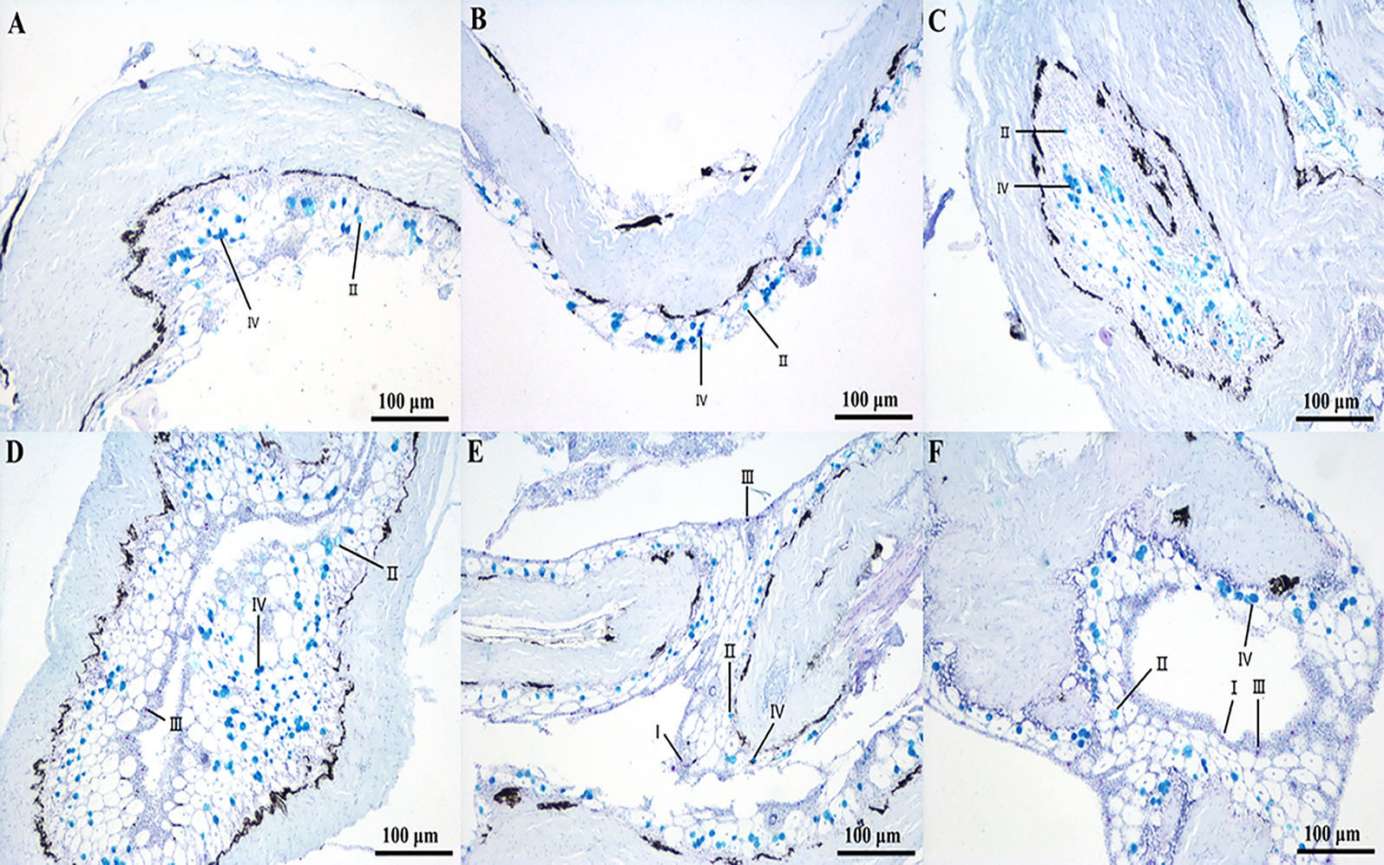
Sections of skin/mucus from fish under transport stress. **(A)** Skin mucous cells of yellow catfish in control group; **(B)** Skin mucous cells of yellow catfish at 0 h transport; **(C)** Skin mucous cells of yellow catfish at 2 h transport; **(D)** Skin mucous cells of yellow catfish at 4 h transport; **(E)** Skin mucous cells of yellow catfish at 8 h transport; **(F)** Skin mucous cells of yellow catfish at 16 h transport.

The mucous cells were stained four different colors by AB-PAS. The results revealed that the skin mucus contained mainly type II and IV mucous cells, and the number of mucus cells was higher in the transportation groups than in the control groups by 4 h. However, after 8 h, the density of mucus cells decreased significantly. The mucus cells were types II and IV at 0 h, 2 h, and 4 h of transport but showed increased proportions of types I and III after 4 h of transport (**Figure 4**).

### 3.4 Analysis and Verification of DEGs

The skin transcriptome data of yellow catfish have been submitted to NCBI GenBank under the accession number GSE179444.

#### 3.4.1 Sequencing of mRNA Libraries

We established and sequenced six mRNA libraries from fish in the control and the 16-h transport stress groups (con-s-1, con-s-2, con-s-3, str-s-1, str-s-2 and str-s-3). The biological replicates had good repeatability. After removing low-quality raw sequences, there were 45216860, 39452880, 44283624, 41127180, 44874282, and 49204244 clean reads in the con-s-1, con-s-2, con-s-3, str-s-1, str-s-2, and str-s-3 libraries, respectively (96.57%–97.09% valid data; Q20 values of 99.97%–99.98%; Q30 values of 98.04%–98.34%, and GC contents of 46%–47%) (**Table 2**). The number of reads that mapped to the yellow catfish genome was 35773763 (con-s-1), 30021210 (con-s-2), 32722551 (con-s-3), 30120316 (str-s-1), 33461205 (str-s-2), and 36262252 (str-s-3). The specific results were listed in **Table 2**.

**Table 2:** Overview of reads for mRNA-seq and quality filtering.

Sample	Raw Data		Valid Data		Valid Ratio	Q20%	Q30%	GC %
	Read	Base	Read	Base				
Con_S_1	46571252	6.99G	45216860	6.78G	97.09	99.98	98.34	47
Con_S_2	40680554	6.10G	39452880	5.92G	96.98	99.98	98.16	47
Con_S_3	45752512	6.86G	44283624	6.64G	96.79	99.97	98.14	46
Str_S_1	42414122	6.36G	41127180	6.17G	96.97	99.97	98.08	46
Str_S_2	46469274	6.97G	44874282	6.73G	96.57	99.97	98.17	46
Str_S_3	50759892	7.61G	49204244	7.38G	96.94	99.97	98.04	46

#### 3.4.2 Identification of DEGs

**Figure 5:**
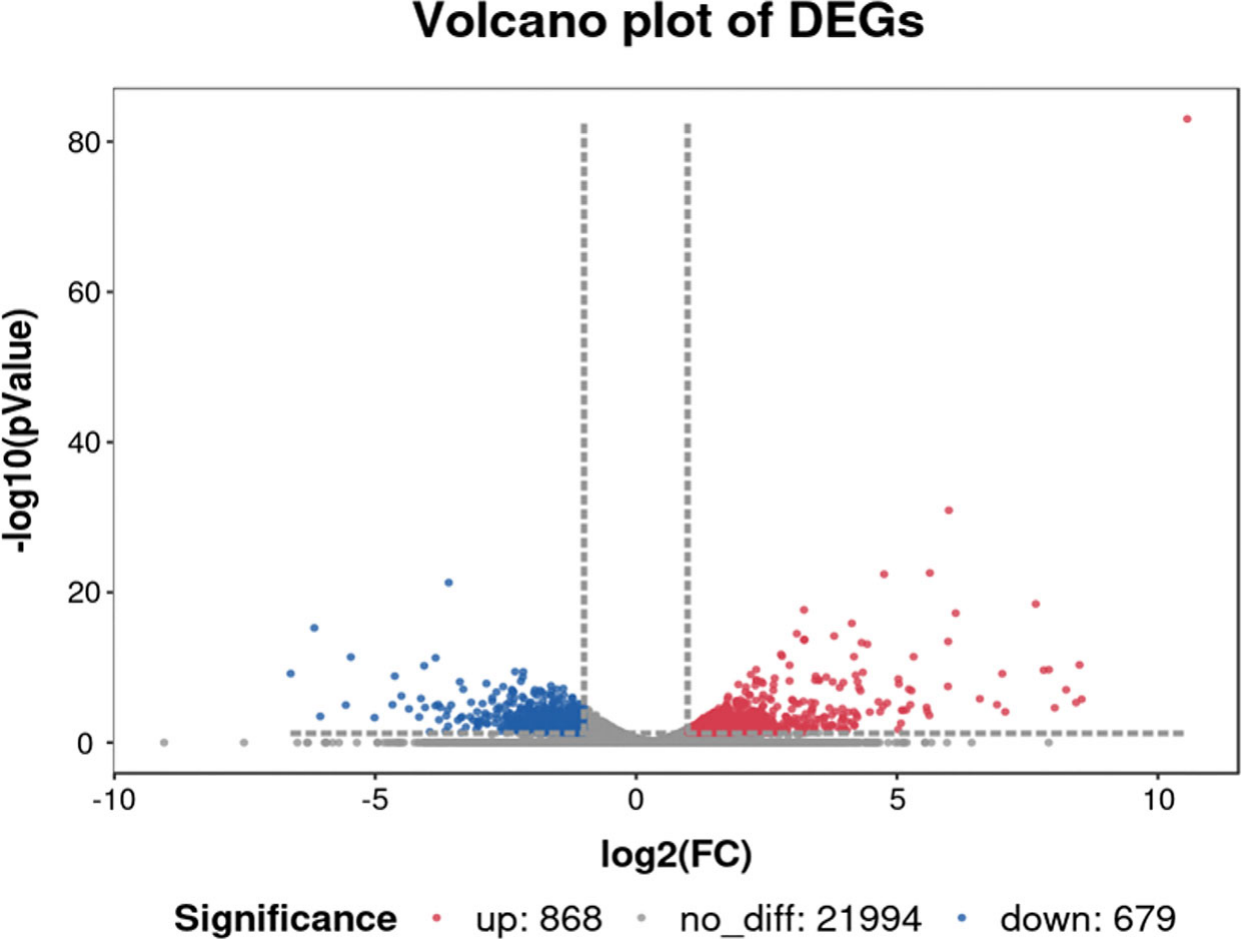
Volcano plot of differentially expressed genes in yellow catfish under transport stress *vs.* yellow catfish in control group. Red and blue dots represent upregulated and downregulated DEGs in transport group compared with control group, respectively. Gray dots represent genes showing no significant differences in expression.

We compared and analyzed the transcriptome data to identify the genes with significant differences in transcript levels between the con-s and str-s libraries, using the following criteria: |log2foldchange|≥1, *P*<0.05, and FPKM>10.We identified 1547 DEGs between the con-s and str-s libraries (868 up-regulated and 679 down-regulated) (**Figure 5**).

#### 3.4.3 GO Classification and KEGG Enrichment Analysis of DEGs

**Figure 6:**
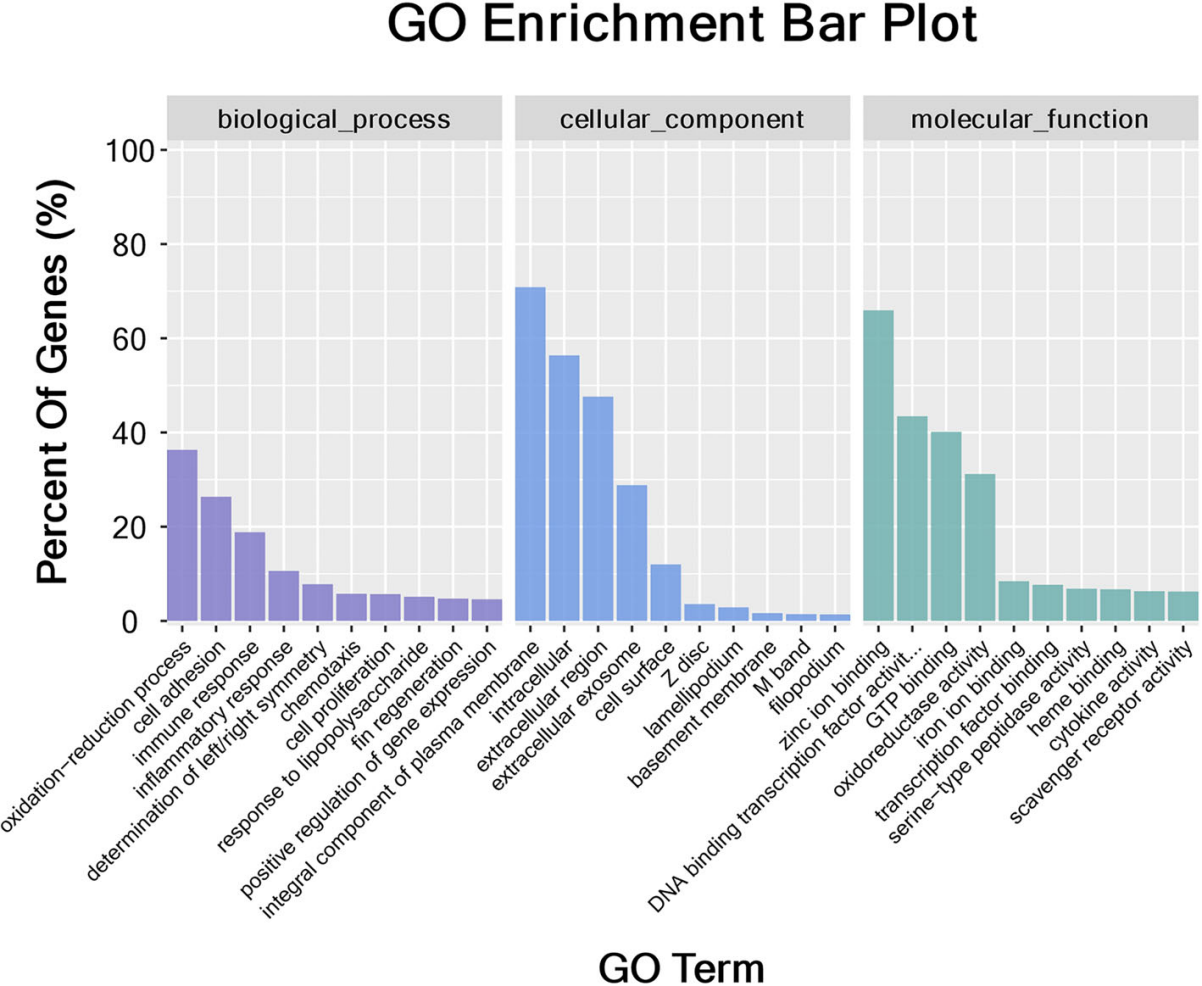
Gene Ontology (GO) categorization of differentially expressed genes in skin. Each annotated sequence was assigned at least one GO term in the following categories: biological process, cellular component, or molecular function.

**Figure 7:**
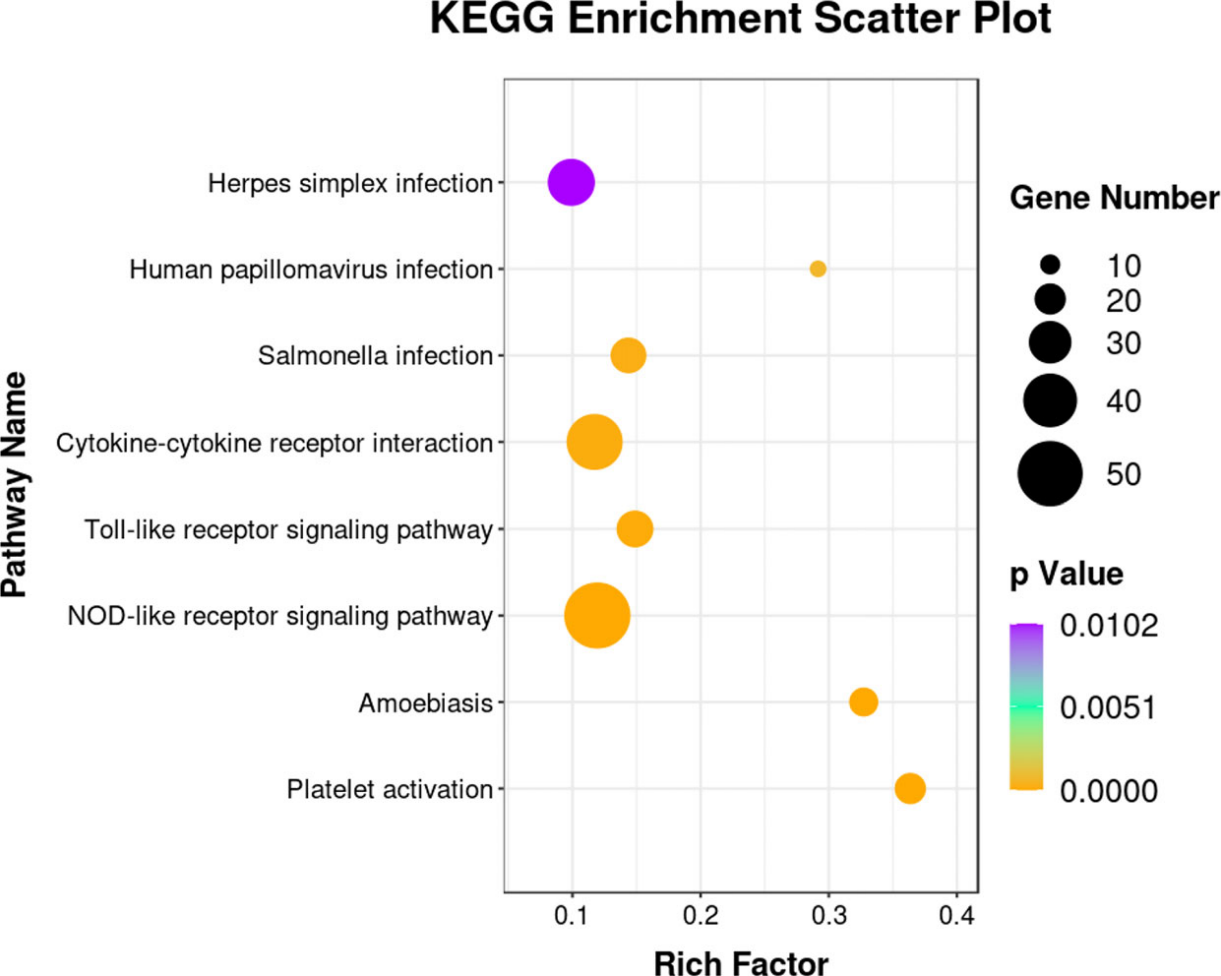
Kyoto Encyclopedia of Genes and Genomes (KEGG) enrichment analysis of immune-associated pathways showing significant differences between control and 16-h transport group.

The DEGs were classified into biological process, cellular component, and molecular function categories by a Gene Ontology enrichment analysis. The GO category enrichment analysis showed that the cellular component subcategories most enriched with DEGs were membrane, integral component of membranes, and nucleus. In the biological process category, the subcategories most enriched with DEGs were biological process, regulation of transcription, and DNA-template. In the molecular function category, the subcategories most enriched with DEGs were metal ion binding and molecular function (**Figure 6**). The sequences of the DEGs were annotated using tools at the KEGG database. The KEGG pathway analysis revealed the pathways most enriched with DEGs in the skin of yellow catfish under transport stress (**Figure 7**), which included the Toll-like receptors (TLRs) signaling pathway, the NOD-like receptors (NLRs) signaling pathway, salmonella infection, cytokine-cytokine receptor interaction, and the AGE-RAGE signaling pathway related to diabetic complications.

#### 3.4.4 Confirmation of Immune-Related DEGs by qRT-PCR

**Figure 8:**
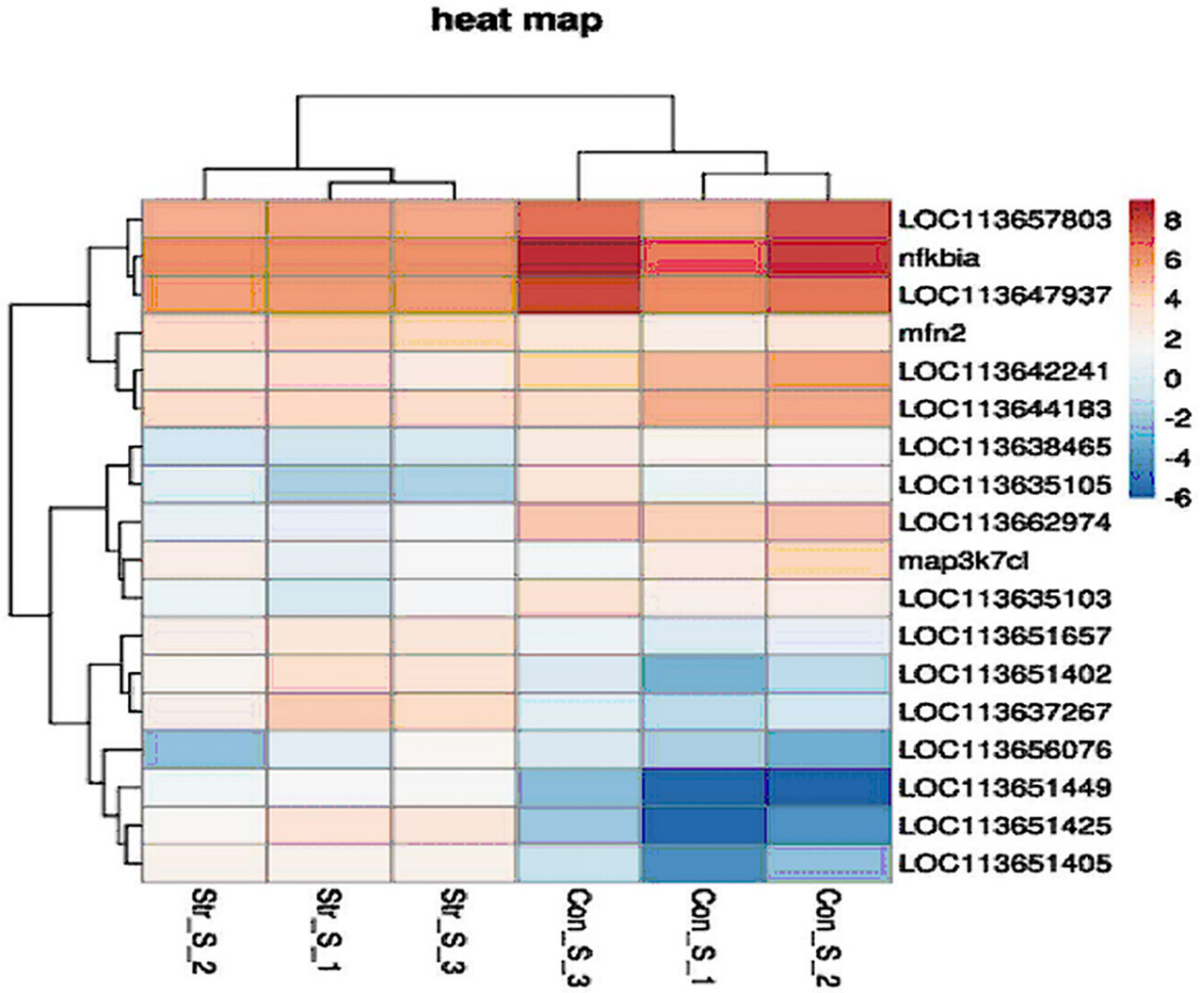
Heat map of 20 selected differentially expressed genes (DEGs) in TLRs/NLRs signaling pathway in skin of hybrid yellow catfish under transport stress (criteria for identification of DEGs: log2 FC > 1 or < -1 and *P* < 0.05). Highly expressed genes are shown in red; genes expressed at low levels are shown in dark blue.

**Figure 9:**
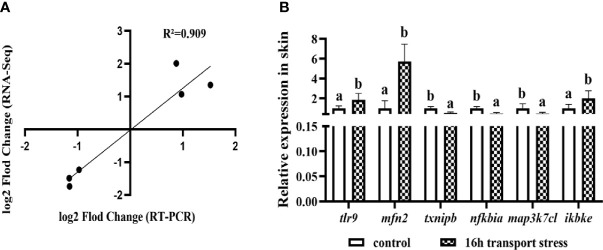
Correlation between transcriptome results and RT-PCR results **(A)**; Validation of *tlr9*, *mfn2*, *txnipb*, *nfkbia*, *map3k7cl* and *ikbke* gene transcript levels in skin by qRT-PCR **(B)**. Values are mean ± SD (*n* =9). Different lowercase letters indicate significant differences (*P* < 0.05).

The 20 DEGs in the TLRs/NLRs signal pathway are shown in a heat map analysis (**Figure 8**). To confirm the reliability of the RNA-Seq data, we selected six immune-related genes for analysis by qRT-PCR. The selected genes were *tlr9*, encoding toll like receptor 9; *mfn2*, encoding mitofusin; *txnipb*, encoding thioredoxin interacting protein b; *nfkbia*, encoding NFKB inhibitor alpha; *map3k7cl*, encoding MAP3K7C-terminal like protein; and *ikbke*, encoding inhibitor of nuclear factor kappa B kinase subunit epsilon. All of these genes are related to the TLRs/NLRs signal pathways. The transcriptome sequencing results of these genes are shown in **Table 3**. Compared with the control group, the 16-h transportation group showed significantly higher transcript levels of *tlr9*, *mfn2* and *ikbke* genes in the skin (*P*<0.05; **Figure 9**) and significantly lower transcript levels of *txnipb*, *nfkbia* and *map3k7cl* (*P*<0.05; **Figure 9B**). The correlation between transcriptome sequencing results and qRT-PCR results is shown in **Figure 9A**.

**Table 3:** Differentially expressed mRNA verified by mRNA-Seq.

Gene abbreviation	Gene description	Log2 (flod-change)	Regulation (HTS vs CO)
*tlr9*	toll like receptor 9	2.01	up
*mfn2*	mitofusins	1.35	up
*txnipb*	thioredoxin interacting protein b	-1.23	down
*nfkbia*	NFKB inhibitor alpha	-1.74	down
*map3k7cl*	MAP3K7 C-terminal like	-1.49	down
*ikbke*	inhibitor of nuclear factor kappa B kinase subunit epsilon	1.07	up

Fold change= Transport group(mean)/Control group (mean),where “mean” is the mean of three biological replicates.

## 4 Discussion

Previous studies have shown that transportation stress can induce an immune response and impair the health of fish (33). It is very important to explore the mechanism of how transport stress effects immune response. During transportation, there are many factors that impose stress upon fish. Experiments using simulated transport treatments have shown that DO, TAN, and NO_2_-N are important water parameters to be managed under transport stress (34). Changes in water quality during transportation can cause stress responses in fish. The concentration of TAN, the major excretory product of fish, in water is an important indicator of fish survival during transport (35). Fish were found to excrete high levels of un-ionized ammonia under transport stress, resulting in increased blood ammonia concentrations and reduced blood oxygen-carrying capacity (36, 37). Increases in the TAN level promote the production of NO_3_^−^, which can be transformed into toxic derivatives, alter physiological functions, and elicit an immune response in the body (38). In the present study, the concentrations of TAN and NO_2_-N in the water increased as the transportation time increased. The highest TAN and NO_2_-N levels were 0.394 mg l^−1^ and 0.123 mg l^−1^, respectively, in the 16h transport stress group. These compounds may have been among the factors that induced the immune response in fish under transport stress (6). Similarly, an inhibitory effect of high concentrations of ammonia on the innate immune response was detected in zebrafish during acclimation to transport-associated stress (25). Additionally, transport-induced changes in stress hormones can also affect the immune function of vertebrates. The way that stressors modify immune function is very complex (39), so further studies are required to determine the mechanism by which transport stress affects the immune response.

Innate immunity is the main immune system of teleost fish due to the underdevelopment of adaptive immunity (40, 41). In the innate immunity of fish, the TLRs/NLRs signaling pathways are the two most widely studied pattern recognition receptors (42). The TLRs are type I transmembrane proteins that can be classified into two groups: cell surface TLRs and intracellular TLRs. The NLRs are intracellular cytoplasmic receptors (43, 44). Transcription of the genes encoding components of the TLRs/NLRs pathways is induced by stress, leading to stimulation of the host’s immune and inflammation responses (45–47). Studies have demonstrated that the activation of the TLR9 pathway provides an important link between immune and inflammatory phenotypes (48, 49). In juvenile turbot, inflammation and the immune response were found to be related to increased expression of genes encoding TLRs, including *tlr2*, *tlr3*, *tlr5b*, *tlr9*, *tlr21*, and *tlr22* (50). In rainbow trout, mechanical injury of muscle tissue was found to trigger the expression of immune-related genes, especially *tlr9*, which showed significantly increased transcript levels at 4 h and 24 h after injury (2.5-fold and 7.2-fold increases, respectively) (51). In our study, we also detected increased transcript levels of *tlr9* in yellow catfish skin at 16 h of transport stress. The transcript level of *mfn2* and *txnipb*, encoding components of the NLRs signaling pathway, were up-regulated and down-regulated respectively in the skin of fish under transport stress. *mfn2* encodes a multifunctional mitochondrial fusion protein that is involved in the activation of innate immunity. This protein can exacerbate inflammation, while knockdown of *mfn2* was shown to ameliorate inflammation (52, 53). Similar to our results, another study found that *mfn2* transcript levels were elevated in human and rat chondrocytes during metabolic disorders and inflammation (52). The *txnipb* plays an important role in pathophysiological consequences including elevated inflammatory response, cellular immunity, and tumorigenesis. Therefore, we speculate that transport stress might induce an immune response and aggravate inflammation through *tlr9*, *mfn2* and *txnipb* mediated TLRs/NLRs pathways. Furthermore, activation of *tlr9* could lead to the recruitment of MyD88, resulting in the activation of NF-κB and MAPK kinases (50). In our study, we detected significantly decreased transcript levels of *nfkbia* and *map3k7cl*, which also encode components of the TLRs/NLRs pathway, in the skin of fish at 16 h of simulated transport. The protein encoded by *nfkbia* was identified as a target for glucocorticoid-mediated immunosuppression (54). In mice, *nfkbia* was poorly expressed and the NF-κB signaling pathway was activated during myocardial infarction, while overexpression of *nfkbia* reduced the expression of inflammatory cytokines and cardiomyocyte apoptosis, and improved cardiomyocyte viability (55). Because *map3k7cl* was downregulated in Chinese patients with non-small cell lung cancer, Niu et al. speculated that *map3k7cl* in the leukocytes may contribute to pathogenesis *via* reducing inflammatory processes, regulating mitochondrial reactive oxygen species signaling, and mediating the Wnt pathway (56). Studies have shown that hyper-activated or incomplete knock-down of TLRs can lead to tissue injury and inflammatory diseases (57). The up-regulated expression of *ikbke* is also associated with the inflammatory response. In infected erythrocytes with low pathogenic avian influenza virus, the expression levels of MyD88, CCL5, and IKBKE were found to be increased (58). In summary, we conclude that 16 h of simulated transport stress induced skin innate immune responses and exacerbated inflammation.

Fish immunity is associated with stronger mucosal defenses. The immune-related components of skin mucus such as LZM, IgM, and alkaline phosphatase play important roles in non-specific immunity (59). As a key enzyme involved in innate immunity, LZM plays a role in defense against pathogens during the early developmental stages of bony fish (60). Its activity varies with fish age, size, and sex, and with the season, temperature, water quality, toxins, and different types of stress (61–64). During the transport of yellow catfish, the activity of LZM in skin mucus might be related to the degree of stress and its duration (65). Previous research showed that in the serum LZM activity was significantly decreased in rainbow trout under transport or water pollution stress (66). IgM is the functional immunoglobulin that is essential for defense mechanisms in the mucosal immunity of fish. It is a common practice to add immunostimulants to enhance innate immune responses, including LZM activity and IgM levels, to relieve stress in fish during transport (67). In the present study, we found that LZM activity and IgM contents peaked at 8 h of transport, and then decreased. This is consistent with the results of Wu et al. (67), who found that LZM activity in the skin mucus and IgM content in the serum tiger grouper first increased and then decreased during transport. The histological results were consistent with these results, and showed that the number and types of mucous cells changed with increasing transport time. The skin mucus cell can mirror the health status of fish. It is a valuable matrix for monitoring stress, pathogen exposure, and nutritional effects. Skin mucus secretion, and components of the mucus, change when aquatic animals are under stress conditions. For example, prolonged exposure to high temperature resulted in an increase and then a decrease in the secretion of skin mucus in yellow croaker (*Larimichthys crocea*) (23). Similarly, a significant increase in mucus secretion was detected in gilthead seabream (*Sparus aurata* L.) under acute crowding and anesthetic stress (68). In sea bass, compared with fish in a control group, those exposed to hypersaline conditions secreted more skin mucus with higher contents of cortisol, glucose, and protein (69). Skin mucus can protect fish against harmful environmental factors and prevent the loss of physiological metabolites and water (70). Increased mucus secretion is a protective mechanism of fish under stress, and is accompanied by enhanced immune function. However, when the degree of the stress exceeds the tolerance range, it can result in immune suppression and decreased mucus secretion. Further research is required to explore the functions of different mucous cell types. At the same time, investigating the difference in mucus production between normal and stressed fish is very important to improve their survival (71). These results may indicate that high-intensity transport stress partially disrupts skin mucosal immune function and causes immunosuppression. After 96 h of recovery, LZM activity and IgM levels were lower than those at 16 h of transport, but still higher than those in the control group, indicating that the immune function of fish skin mucus was still recovering. Notably, some studies have shown that teleost mucosal IgM is generated independently of systemic antibody (72). Therefore, there may be weak relationships between the tissue immune system and the mucosal cell immune system (73).

Blood is also an important component of the immune system, and changes in hematological parameters can be indicative of the physiological health of fish (74, 75). Under stress conditions, changes in the skin immunity of fish are often accompanied by changes in various hematological indices (76). The WBC, RBC, HGB, and HCT are the hematological variables that are most commonly monitored during stress (77). Ronza et al. (78) confirmed that RBC have important immunological functions through a blood transcriptomics analysis of healthy and diseased turbot (*Scophthalmus maximus*). WBC are involved in cellular immunity, an increase in the WBC count is often correlated with increased antibody production (79). Thus, on the basis of our results, we speculate that the increase in the number or specific volume of RBC and WBC was due to the transport stress-induced enhanced hematological-immunological response. Consistent with our results, Boaventura et al. (80) reported increased HGB, HCT, and total plasma protein in juveniles of *Lophiosilurus alexandri* after a 4-h transport treatment. Other studies have reported that exposure to ammonia and nitrite can lead to decreases in WBC, RBC, HGB, and HCT in *Takifugu rubripes* and yellow catfish (81, 82). Therefore, the changes in the number or specific volume of RBC and WBC at 16 h of transport in this study may be due to immunosuppression caused by the increased contents of TAN and NO_2_-N in the water during a long transport period. After 96 h of recovery, the amount of RBC and the levels of HGB and HCT returned to normal, but the amount of WBC remained higher than that in the control group. This might indicate that blood immunity was not fully restored after transport stress. The modulation of blood parameters during stress is related to splenic contraction and hemodilution. The sustained contraction of the spleen increases the amounts of circulating WBC, RBC, and HGB in the blood (83–85). Therefore, the changes in hematological immunity are probably related to splenic contraction and changes in blood parameters that were secondary to transport stress.

In summary, we explored the effects of transport stress on yellow catfish by conducting physiological, histological, and molecular analyses of the skin. During the transport of hybrid yellow catfish, TAN and NO_2_^-^N accumulated in the water, and this may be one of the factors that triggered the immune response in the skin. On the one hand, the skin transcriptome results suggested that 16 h of transport stress may induce immune responses and stimulate pro-inflammatory responses by up-regulating *tlr9*, *mfn2*, and *ikbke* and down-regulating *txnipb*, *nfkbia* and *mapk3cl* in the TLRs/NLRs pathways. On the other hand, transport times shorter than 8 h may not seriously damage the mucous immune system, but longer transport times (16 h) may inhibit the immune function of mucus, leading to the decrease of LZM and IgM, and the change of the number and type of mucus cells. In addition, on the basis of the performance of the recovery group, we suggest that the physiological recovery time should be longer than 96 h after 16 h of transport stress.

Minimizing the effects of stress during transport of hybrid yellow catfish is important for successful aquaculture. In addition, a better understanding of the stress response of hybrid yellow catfish to transport is necessary to formulate optimal transport procedures. On the basis of our research, we identified two possible ways to reduce transport stress. Firstly, inadequate or excessive mucus secretion is not conducive to fish survival, so controlling mucus secretion during transport will improve the survival rate (71). Secondly, total ammonia–nitrogen and nitrite–nitrogen can cause immunosuppression, so controlling their levels in the water during transport will also reduce the adverse effects of transport stress.

## Data Availability Statement

The datasets presented in this study can be found in online repositories. The names of the repository/repositories and accession number(s) can be found in the article/supplementary material.

## Ethics Statement

The animal study was reviewed and approved by Freshwater Fisheries Research Center at the Chinese Academy of Fishery Sciences (Wuxi, China). Written informed consent was obtained from the owners for the participation of their animals in this study.

## Author Contributions

TZ: Investigation, Methodology, Writing - original draft, Formal analysis. ZS: Methodology, Supervision. JQ: Formal analysis, Data curation, Writing - review & editing. YT: Formal analysis, Investigation, Methodology, Resources. HZ: Investigation, Validation. PX: Supervision, Funding acquisition, Writing - review & editing. All authors contributed to the article and approved the submitted version.

## Funding

This research was supported by the Natural Science Foundation of Jiangsu Province, China (Grant no. BK20181137), Project of Six Talent Peaks in Jiangsu Province (NY-133).

## Conflict of Interest

The authors declare that the research was conducted in the absence of any commercial or financial relationships that could be construed as a potential conflict of interest.

## Publisher’s Note

All claims expressed in this article are solely those of the authors and do not necessarily represent those of their affiliated organizations, or those of the publisher, the editors and the reviewers. Any product that may be evaluated in this article, or claim that may be made by its manufacturer, is not guaranteed or endorsed by the publisher.
